# Perinatal outcomes of singletons following double vitrification-warming procedures: a retrospective study using propensity score analysis

**DOI:** 10.1186/s12884-023-05369-z

**Published:** 2023-01-14

**Authors:** Xiaoyue Shen, Min Ding, Yuan Yan, Chenyang Huang, Shanshan Wang, Jianjun Zhou, Jun Xing

**Affiliations:** Reproductive Medicine Center, Drum Tower Hospital Affiliated to Nanjing University Medical School, Zhongshan Road 321, Nanjing, 210008 China

**Keywords:** Vitrification, Cryopreservation, Frozen-thawed embryo transfer, Birth weight, Large for gestational age

## Abstract

**Background:**

Although repeated cryopreservation is an occasional occurrence, the effect on perinatal outcomes is unclear. Therefore, the aim of this study was to evaluate the perinatal outcomes of singletons after embryo re-cryopreservation.

**Methods:**

In this retrospective study, a total of 647 singleton live births after blastocyst freeze-thaw embryo transfer cycles were investigated. They were divided into two groups: vitrified-warmed blastocysts (once-vitrified group) and vitrified-warmed blastocysts derived from thawed cleaved embryos (re-vitrified group). Propensity score matching (PSM) was used to control for potential confounding factors.

**Results:**

A total of 592 infants were included in the once-vitrified group, and 55 infants were included in the re-vitrified group. After PSM, 108 cases were generated for comparison. The median gestational age was 38 weeks for both groups, and the birthweights were comparable (3390.6 ± 601.5 g vs. 3412.8 ± 672.6 g, *P* > 0.05). The incidence of preterm birth (PTB) (20.4% vs. 16.7%), low birthweight (LBW) (3.7% vs. 7.4%), macrosomia (11.1% vs. 16.7%) and large for gestational age (LGA) (29.6% vs. 22.2%) were not significantly different between the two groups. Logistic regression analysis indicated that double vitrification-warming procedures did not affect the occurrence of PTB (OR, 2.58 [95% CI, 0.77, 8.63]), LBW (OR, 0.83 [95% CI, 0.08, 8.29]), macrosomia (OR, 0.60 [95% CI, 0.13, 2.69]), or LGA (OR, 1.51 [95% CI, 0.53, 4.27]) (*P* > 0.05, for all).

**Conclusion:**

Our findings demonstrate that double vitrification-warming procedures do not increase the risk of adverse neonatal outcomes compared with those of once-vitrified embryos.

**Supplementary Information:**

The online version contains supplementary material available at 10.1186/s12884-023-05369-z.

## Background

It has been decades since the first in vitro fertilization (IVF) infant was born; since then, assisted reproduction has advanced significantly. The number of available embryos in each IVF cycle has also increased, resulting in a slew of surplus of embryos undergoing cryopreservation. Cryopreservation of embryos increases the cumulative pregnancy rate for a single cycle of ovarian stimulation with the clinical pregnancy rate and live birth rate being no lower than in the fresh cycle, while reducing the risk of multiple pregnancies and ovarian stimulation [1–3]. As such, cryopreservation has become a widespread reliable procedure in assisted reproductive technologies [4]. However, many studies have also shown that frozen-thawed embryo transfer (FET) increases the risks of pregnancy-induced hypertension, large for gestational age and high birth weight infants compared with fresh embryo transfer [5–7].

With the implementation of the single embryo transfer strategy, the number of surplus embryos has increased [8]. When more than one embryo was frozen in one cryo-straw but only one embryo was transferred, the surplus embryo will be refrozen. Dose re-cryopreservation have negative effects on clinical outcomes? On present understanding, repeated cryopreservation procedures may reduce embryo implantation rate，clinical pregnancy rate and live birth rate [9, 10]. However, limited published data have focused on the perinatal outcomes of embryos experiencing repeated cryopreservation. Due to heterogeneity between studies, the current conclusions are not clear. Therefore, in order to further clarify whether repeated cryopreservation has adverse effects on offspring, we retrospectively analyzed the perinatal outcome of embryos repeated cryopreservation by vitrification in the present study.

## Methods

### Study design

This retrospective study was approved by the Medical Ethics Committee of Nanjing Drum Tower Hospital affiliated to Nanjing University Medical School on 6 May 2021 (reference number 2021–163-01).

All medical files including frozen-thawed blastocyst cycles from January 2013 to December 2019 at the Reproductive Medicine Centre of Nanjing Drum Tower Hospital were retrospectively reviewed. Inclusion criteria: (1) patients undergoing single blastocyst transfer with live singleton births and known perinatal outcomes; (2) patients have experienced at least one frozen-thawed cycle and did not have a live birth in the preceding cycle. Patients with endometriosis, abnormal uterine pathology or undergoing preimplantation genetic testing or those with history of recurrent pregnancy loss were excluded. In total, 647 live born singletons were included in this study. They were divided into two groups according to the transferred embryo cryopreservation time: vitrified-warmed blastocysts (once-vitrified group, *n* = 592) and vitrified-warmed blastocysts derived from thawed cleaved embryos (re-vitrified group, *n* = 55).

### IVF/ICSI procedure and embryo culture

Depending on the patient’s age, ovarian reserve, and ovarian responses in the previous ovulation cycle, gonadotrophin-releasing hormone (GnRH) agonist protocol or antagonist protocol was used [11].

GnRH-agonist protocol: In mid luteal phase of the preceding cycle, a short-acting GnRH-a (Triptorelin, Ferring AG, Germany) was administered daily for 14 days and follicular ultrasonography, serum luteinizing hormone (LH), follicle stimulating hormone (FSH), estradiol (E_2_) and progesterone were measured. 150–300 IU recombinant FSH (Gonal-F, Merck Serono, Switzerland) was administered daily when the serum FSH and LH levels were < 5 mIU/mL and E_2_ was < 50 pg/mL. GnRH-a was continued until the trigger day. The dosage of recombinant FSH was adjusted according to the ovarian response, and human menopausal gonadotropin (HMG, LIVZON, China) or recombinant LH (Luveris, Merck Serono, Switzerland) was added as needed.

GnRH-antagonist protocol: 150–300 IU recombinant FSH was initiated on Day 2 or 3 of the menstrual cycle until trigger day. The dosage of recombinant FSH was adjusted, and HMG or recombinant LH was added according to the ovarian response evaluated by transvaginal ultrasonography and serum hormone levels. 0.25 mg cetrorelix (Cetrotide, Merck Serono, France) was used daily when the leading follicles reached a mean diameter of 14 mm until trigger day.

For both protocols, if two to three dominant follicles reached a diameter of 18 mm, 250 μg recombinant human chorionic gonadotropin (hCG) (Ovitrelle, Merck Serono, France) was injected. The oocytes were retrieved under transvaginal ultrasound guidance 36–38 hours after recombinant hCG administration. Retrieved oocytes were then fertilized in conventional IVF or intracytoplasmic sperm injection (ICSI). Two pronuclei or two polar bodies can be observed in normal fertilization 16–18 h post insemination. Embryos were cultured in G1/G2 sequential media (Vitrolife, Goteborg, Sweden) at 37 °C in 6% CO_2_, 5% O_2_ and 89% N_2_ high-humidity incubator. Blastocyst morphological evaluation was based on the Gardner scoring system [12].

### Vitrification cryopreservation and thawing procedures

The vitrification cryopreservation and thawing procedures were carried out using a vitrification or warming kit from Kitazato (Tokyo, Japan). Before vitrification, the blastocyst was artificially shrunken by laser drilling. Cleavage-stage embryos or shrunken blastocysts were preequilibrated in equilibration solution for 5–8 min at room temperature and then placed into vitrification solution. One minute later, the embryos were loaded onto the surface of Cryotop (Kitazato, Tokyo, Japan) and then submerged into liquid nitrogen immediately, using open vitrification devices.

The warming procedure was referred to our center’s previous research [13]. The embryo was immediately transferred to preequilibrated thawing solution for 1 min and dilution solution for 3 min and then washed twice in washing solutions 1 and 2 for 5 min each; the embryo was then cultured in a 100 μl droplet of culture medium G2 (Vitrolife, Goteborg, Sweden) overlaid with light paraffin oil (Vitrolife, Goteborg, Sweden) in the incubator at 37 °C in a humidified atmosphere with 5% O_2_, 6% CO_2_ and 89% N_2_ for 2–4 h and then assessed. Laser-assisted hatching was applied to vitrified–warmed blastocysts except for those blastocysts with an expansion degree of V or VI. Generally, blastocysts with re-expanded blastocoel cavities with clear morphology and a bright lustre were considered to have survived.

### Outcome measures

Overall baseline clinical characteristics for each patient included age, body mass index (BMI), basal FSH, duration of infertility, parity, type of infertility and cause of infertility, insemination methods (IVF or ICSI), endometrium preparation protocol for FET, and endometrial thickness.

Any singleton birth ≥24 weeks of gestation was considered a live singleton birth. The birth weight, gestational age at delivery and sex were recorded for all live singletons. The neonatal outcomes evaluated were preterm birth (PTB, delivery between 24 and 37 weeks), low birthweight (LBW, birthweight < 2500 g), macrosomia (birthweight > 4000 g), average birthweight, sex ratio, small for gestational age (SGA), and large for gestational age (LGA). SGA was defined as a weight below the 10th percentile for gestational age, and LGA was defined as having a birthweight greater than the 90th percentile for gestational age at birth [14].

### Statistical analysis

Continuous variables were described by using the mean ± SD or median (interquartile range, IQR), while categorical variables were described as absolute numbers and percentages. Student’s test was used to compare the differences of normally distribution parameters, while for non-normal distribution parameters, Wilcoxon rank sum test were used. Pearson’s chi-squared test or Fisher’s precision probability test was used to compare the differences of categorical variables. To help account for the nonrandomized administration of re-vitrified embryos, we used propensity-score methods to reduce the effects of confounding. Matching was performed with the use of a 1:1 matching protocol without replacement, with a caliper width equal to 0.01 of the standard deviation of the logit of the propensity score. *P* values < 0.05 were considered statistically significant. The variables included maternal age, parity, basal FSH, BMI, duration of infertility, type of infertility, parity, and cause of infertility. To analyse the associations between the cryopreserved time and perinatal outcomes, logistic regression models were conducted for each outcome indicator using the before and after matching data, and odds ratios (OR) and their 95% CI before and after adjusting for confounders were calculated. Statistical analyses were performed using SPSS statistical package version 26.0.

## Results

### Clinical characteristics and outcomes of the study cohort

**Fig. 1:**
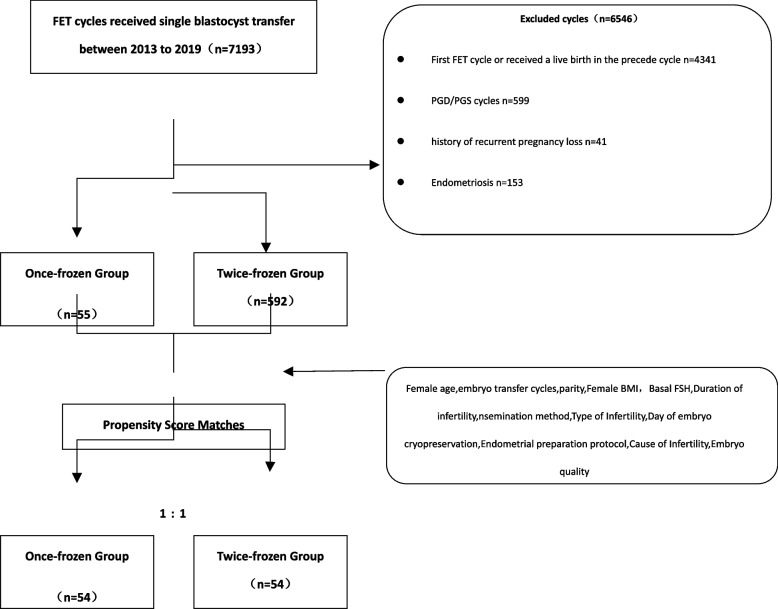
Flowchart of the inclusion and exclusion of participants in this study

A total of 7193 frozen-thawed single blastocyst transfer cycles were reviewed from January 2013 to December 2019 in this retrospective study. According to the inclusion and exclusion criteria, the remaining 647 cycles were analysed (Fig. 1). The patients’ overall baseline demographics, baseline IVF characteristics and perinatal outcomes of the whole study population are displayed in Table 1. The mean female age at retrieval was 30.3 ± 4.4 years. The average BMI and basal FSH of the study population were 22.5 ± 3.3 kg/m^2^ and 7.4 ± 2.7 mIU/mL, respectively. A total of 75.7% of patients underwent IVF, while the remaining 24.3% of patients underwent ICSI. In the FET cycles, hormone-replacement therapy (HRT) was predominantly used for endometrium preparation (83.3%). The average birthweight for singletons was 3361.5 ± 589.3 g. The preterm birth rate in singletons was 15.1%, and the rates of low birthweight and macrosomia were 5.6 and 13%, respectively.

**Table 1:** Overall Baseline clinical characteristics and perinatal outcomes of the study cohort (*n* = 647)

Demographics	
Female age at retrieval, years	30.3 ± 4.4
Parity	
Primipara	405 (62.6)
Multipara	242 (37.4)
Body mass index, kg/m^2^	22.5 ± 3.3
Basal FSH, mIU/mL	7.4 ± 2.7
Duration of infertility, years	3.6 ± 2.7
Type of Infertility	
Primary	316 (48.8)
Secondary	331 (51.2)
Cause of Infertility	
Ovulation disorder	40 (6.2)
Tubal factor	285 (44.0)
Male factors	68 (10.5)
Other factors	254 (39.3)
**IVF characteristics**	
Insemination method	
IVF	490 (75.7)
ICSI	157 (24.3)
Endometrial thickness, mm	9.4 ± 1.7
Endometrial preparation protocol	
HRT	539 (83.3)
Ovulation induction	45 (7.0)
NC	63 (9.7)
**Perinatal outcomes**	
Gestational age, weeks	38 (37–39)
PTB (< 37 weeks)	98 (15.1)
Gender	
Boys	361 (55.8)
Girls	286 (44.2)
Birthweight, g	3361.5 ± 589.3
LBW (< 2500 g)	36 (5.6)
Macrosomia (≥ 4000 g)	84 (13)

Data are presented as mean ± SD, median (IQR) and n (%). *BMI* Body mass index, *NC* Natural cycle, *HRT* Hormone replacement therapy, *PTB* Preterm birth, *LBW* Low birthweight

### Propensity score matching of basic characteristics between the once-vitrified group and the re-vitrified group

Among a total of 647 singleton live births, 55 infants resulted from the re-vitrified group, while 592 were from the once-vitrified group. The comparisons of clinical characteristics between the two groups are listed in Table 2. In the once-vitrified group, most of the patients were primipara (65.9%), and their BMI was higher (22.5 ± 3.4 kg/m^2^ vs. 22.2 ± 2.7 kg/m^2^, *P* = 0.03).

**Table 2:** The propensity score matching of basic characteristics between once-vitrified group and re-vitrified group

	Before matching			After matching		
	Once-vitrified group (*n* = 592)	Re-vitrified group (*n* = 55)	P	Once-vitrified group (*n* = 54)	Re-vitrified group (*n* = 54)	P
Female age at retrieval，years	30.4 ± 4.4	29.3 ± 4.1	0.41	29.6 ± 4.1	29.3 ± 4.2	0.71
Primipara	390 (65.9)	15 (27.3)	<0.001	19 (35.2)	15 (27.8)	0.13
Body mass index, kg/m^2^	22.5 ± 3.4	22.2 ± 2.7	0.03	22.4 ± 3.4	22.2 ± 2.8	0.72
Basal FSH, mIU/mL	7.5 ± 2.8	6.5 ± 1.5	0.06	7.1 ± 3.2	6.6 ± 1.5	0.25
Duration of infertility, years	3.5 ± 2.7	3.7 ± 2.5	0.43	3.8 ± 2.5	3.7 ± 2.5	0.73
Insemination method			0.44			0.82
IVF	446 (75.3)	44 (80.0)		41 (75.9)	42 (79.6)	
ICSI	146 (24.7)	11 (20.0)		13 (24.1)	11 (20.4)	
Type of Infertility			0.08			0.25
Primary	283 (47.8)	33 (60.0)		25 (46.3)	32 (59.3)	
Secondary	309 (52.2)	22 (40.0)		29 (53.7)	22 (40.7)	
Endometrial preparation protocol			0.81			0.57
HRT	491 (82.9)	48 (87.3)		43 (79.6)	47 (87)	
Ovulation induction	42 (7.1)	3 (5.5)		4 (7.4)	3 (5.6)	
NC	59 (10.0)	4 (7.3)		7 (13)	4 (7.4)	
Cause of Infertility			0.15			0.40
Ovulation disorder	38 (6.4)	2 (3.6)		3 (5.6)	2 (3.7)	
Tubal factor	253 (42.7)	32 (58.2)		26 (48.1)	31 (57.4)	
Male factors	62 (10.5)	6 (10.9)		3 (5.6)	6 (11.1)	
Other factors	239 (40.4)	15 (27.3)		22 (40.7)	15 (27.8)	
Embryo quality			0.23			0.51
High quality embryos	405 (68.4)	42 (76.4)		38 (70.4)	42 (77.8)	
Good quality embryos	187 (31.6)	13 (23.6)		16 (29.6)	12 (22.2)	

Data are presented as mean ± SD and n (%). *NC* Natural cycle, *HRT* Hormone replacement therapy. The blastocyst score was determined using Gardner blastocyst grading scale. Both the blastocyst inner cell mass and the trophectoderm cell cluster rated ≥B as high quality blastocysts; either the blastocyst inner cell mass or the trophectoderm cell cluster rated C as good quality blastocysts

To eliminate the influence of baseline characteristics on outcomes, 1:1 propensity score matching was performed between the two groups. Finally, a total of 108 cases were matched by their propensity score. Before propensity score matching, the two groups had significantly different characteristics, but propensity score matching balanced characteristics between the cohorts (Table 2), suggesting that the matched cohorts had highly similar baseline characteristics.

### Perinatal outcomes between the once-vitrified group and re-vitrified group before and after matching

The perinatal outcomes are summarized in Table 3. No difference was observed in birthweights between the two groups before or after matching (3356.8 ± 582.1 g vs. 3412.6 ± 666.4 g，3390.6 ± 601.5 g vs. 3412.8 ± 672.6 g). In the once-vitrified group, before or after matching LBW and macrosomia were comparable to those in the re-vitrified group (*P* > 0.05). After matching，PTB occurred in 20.4 and 16.7% of the once-vitrified and re-vitrified groups, respectively, without a significant difference. Likewise, there was no significant difference in the incidence of SGA or LGA between the two groups (P > 0.05). No differences were observed in the percentage of boys born between the two matched cohorts (61.1% vs. 51.9%, P > 0.05). In order to eliminate gender bias on birth weight，we further analyzed the birth weight of boys and girls respectively, and found that birthweight outcomes was comparable between once-vitrified group and re-vitrified group in either male or female neonates. Maternal complications may also adversely affect perinatal outcomes, so we analyzed the incidence of maternal complications. As shown in supplemental Table 1, there was no difference in the incidence of maternal complications between the two groups.

**Table 3:** Perinatal outcomes of once-vitrified group and re-vitrified group before and after PS matching

	Before matching			After matching		
	Once-vitrified group (*n* = 592)	re-vitrified group (*n* = 55)	P	Once-vitrified group(*n* = 54)	re-vitrified group (*n* = 54)	P
Gestational age, weeks	38 (37–39)	38 (37–39)	0.92	38 (37–39)	38 (37.7–39)	0.09
PTB (< 37 weeks)	89 (15.0)	9 (16.4)	0.79	11 (20.4)	9 (16.7)	0.80
Neonatal abnormalities	7 (1.2)	0 (0.00)	1.00	0 (0.0)	0 (0.00)	/
Birthweight, g	3356.8 ± 582.1	3412.6 ± 666.4	0.87	3390.6 ± 601.5	3412.8 ± 672.6	0.86
LBW (< 2500 g)	32 (5.4)	4 (7.3)	0.56	2 (3.7)	4 (7.4)	0.67
Macrosomia (≥ 4000 g)	75 (12.7)	9 (16.4)	0.44	6 (11.1)	9 (16.7)	0.58
LGA	134 (22.6)	13 (23.6)	0.86	16 (29.6)	12 (22.2)	0.38
SGA	15 (2.5)	0 (0)	0.63	1 (1.9)	0 (0)	0.49
Gender			0.45			0.44
Boy	333 (56.3)	28 (50.9)		33 (61.1)	28 (51.9)	
Girl	259 (43.7)	27 (49.1)		21 (38.9)	26 (48.1)	
Boys						
Gestational age, weeks	38 (37–39)	38 (37–39)	0.48	37 (37–38.5)	38 (37–39)	0.36
Birthweight, g	3406.5 ± 585.4	3459.3 ± 792.4	0.09	3479.1 ± 593.1	3459.3 ± 792.4	0.17
LBW (< 2500 g)	17 (5.1)	3 (10.7)	0.20	1 (3.0)	3 (10.7)	0.32
Macrosomia (≥ 4000 g)	50 (15.0)	6 (21.4)	0.37	4 (12.1)	6 (21.4)	0.53
Girls						
Gestational age, weeks	38 (37–39)	38 (37–39)	0.59	38 (36.5–39)	38 (38–39)	0.23
Birthweight, g	3292.8 ± 572.5	3364.2 ± 515.2	0.14	3251.4 ± 602.2	3362.8 ± 525.3	0.32
LBW (< 2500 g)	15 (5.8)	1 (3.7)	1.00	1 (4.8)	1 (1.8)	1.00
Macrosomia (≥ 4000 g)	25 (9.6)	3 (11.1)	0.74	2 (9.5)	3 (11.5)	1.00

Data are presented as mean ± SD, median (IQR) and n (%). *PTB* Preterm birth, *LBW* Low birthweight, *LGA* Large for gestational age, *SGA* Small for gestational age

### Modification of the cryopreservation time effect on perinatal outcomes by logistic regression models

We used logistic regression models to demonstrate the effect of double vitrification-warming procedures on perinatal outcomes. As seen in Table 4, compared to the once-vitrified group, the ORs for PTB, LBW, macrosomia and LGA in the re-vitrified group were 0.90 (95% CI: 0.43, 1.91), 0.73 (95% CI: 0.25, 2.14), 0.74 (95% CI: 0.35, 1.58) and 0.94 (95% CI: 0.49, 1.81), respectively, with *P* > 0.05, before matching. After matching, the risks of PTB, LBW, macrosomia and LGA in the re-vitrified group compared to the once-vitrified group were 1.28 (95% CI: 0.48, 3.39), 0.48 (95% CI: 0.08, 2.74), 0.63 (95% CI: 0.21, 1.90) and 1.47 (95% CI: 0.62, 3.51), respectively. The adjusted outcome showed that PTB, LBW, macrosomia, LGA and birthweight were not significantly different when adjusted for all confounding factors, including gestational age and infant sex.

**Table 4:** Adjusted odds ratios of PTB, LBW, macrosomia and birthweight difference (once = 1)

Embryo vitrification-warming times	PTB		LBW		Macrosomia		Birthweight		LGA	
	OR(95%CI)	P	OR(95%CI)	P	OR(95%CI)	P	β(95%CI)	P	OR(95%CI)	P
Unadjusted OR before matching (*n* = 647)	0.90 (0.43, 1.91)	0.79	0.73 (0.25, 2.14)	0.56	0.74 (0.35, 1.58)	0.44	55.81 (− 107.39, 219.01)	0.50	0.94 (0.49, 1.81)	0.86
Adjusted OR before matching (*n* = 647)	1.06 (0.47, 2.40)	0.88	0.77 (0.05, 12.32)	0.85	0.67 (0.29, 1.59)	0.37	40.11 (−81.66, 210.17)	0.52	1.03 (0.50, 2.11)	0.93
OR after matching (*n* = 108)	1.28 (0.48, 3.39)	0.62	0.48 (0.08, 2.74)	0.41	0.63 (0.21, 1.90)	0.41	22.28 (− 221.17, 265.72)	0.86	1.47 (0.62, 3.51)	0.38
Adjusted after matching (*n* = 108)	2.58 (0.77, 8.63)	0.12	0.83 (0.08, 8.29)	0.88	0.60 (0.13, 2.69)	0.51	1.51 (− 187.74, 190.77)	0.99	1.51 (0.53, 4.27)	0.44

Adjusting for female retrieval age, BMI, FSH, embryo transfer cycles, parity, the type of infertility and the quality of embryos, gestational age and infant gender. β is the regression coefficient or partial regression coefficient of linear regression

## Discussion

More and more children are born using assisted reproductive technology (ART) globally, so the safety of offspring is increasingly concerned. In this retrospective study, we assessed the effect of repeated vitrification-warming procedures on perinatal outcomes of human embryos. According to our research results, no poor outcomes were observed among singletons following repeated vitrification-warming procedures compared to once vitrification-warming procedures.

Embryo cryopreservation is a crucial part of ART. Compared with fresh embryo transfer, frozen embryo transfer is associated with a higher risk of LGA and higher birth weight in singletons [6, 15]. However, the safety of offspring after repeated frozen embryo transfer has not been widely reported at present. After all, re-cryopreservation of embryos is an occasional event. There were several case reports demonstrating healthy live births after frozen-thawed embryo transfer cycles with embryos that were frozen-thawed twice [16–18]. And some early retrospective studies have also shown that the embryos re-cryopreservation does not affect clinical pregnancy rates [19, 20]. However, other recent studies have shown that transfer of twice frozen-thawed embryos increase the rate of miscarriage and reduce the clinical pregnancy rate and live birth rate [9, 10, 21]. The discrepancies in the results of these studies may be due to the different methods used to freeze embryos，embryos frozen at different stages and the differences in baseline characteristics of the study population. Furthermore, these studies may exist the sample selection biases, as the patients underwent repeated cryopreserved embryo transfer may always experience previous embryo transfer failure, so the control group should be consistent with this. The first follow-up study to present perinatal outcomes of children born after embryo re-cryopreservation showed that human refrozen-thawed embryos resulted in normal live births after FET at a similar rate to that of once-frozen-thawed embryos [22]; however, detailed neonatal outcomes were not described. Two other studies reported that repeated cryopreservation of embryos have no negative impact on neonatal outcomes [9, 21], but they did not eliminate the possible bias of confounding factors on the results.

In the present study, patients who had experienced previous embryo transfer failure were set as the once-vitrified group, and all embryos were frozen using the same vitrification method. Furthermore, to eliminate the influence of clinical baseline characteristics on the results, a propensity score matching, and logistic regression were used to control for potential confounding variables. After PSM, the incidence of PTB, LBW, macrosomia and LGA was not different between the once-vitrified group and the re-vitrified group. Logistic regression after adjusting for relevant confounding factors showed that the number of vitrification-warming procedures was not related to the incidence of PTB, LBW, macrosomia, LGA or birthweight.

It has been reported that the effect of FET on birthweight may interact with sex [7, 23]. Litzky et al.’s study indicated that FET influenced male birthweight, but it did not have effects on female birthweight [7]. To eliminate this important confounder, we performed sex-stratified analyses to improve interpretability in our study. There was no significant difference in birthweight, LBW, or macrosomia in either male or female neonates. In addition, perinatal outcomes are also closely related to maternal obstetric complications. For singletons, the risk of hypertensive disorders after FET was higher than after fresh cycles or spontaneous pregnancies [24, 25]. The increased risk of hypertensive disorders among FET may be related to alteration in methylation of regulatory genes involved with implantation or to programmed FET protocols [26, 27]. Whereas the association of FET with GDM is deficient in previous studies [28–30]. Studies have shown that hypertensive disorders of pregnancy increase the prevalence of low birth weight [31], and gestational diabetes mellitus (GDM) increases the incidence of macrosomia [32, 33]. Consequently, we further analysed maternal complications in the once-vitrified group and re-vitrified group. There was no difference in gestational hypertension or gestational diabetes before and after data matching. Also, other maternal complications did not differ between the two groups, as shown in Supplemental Table 1. Therefore, the bias caused by maternal complications during pregnancy can be excluded.

Of course, there are several limitations in our study. First, it was a retrospective study with single-centre participation. Second, although we used propensity score matching to eliminate the influence of baseline characteristics on FET outcomes as much as possible, it could not eliminate all bias caused by confounders between the two groups. In addition, because of the limited sample size, it was difficult to stratify the effects of repeated vitrification-warming procedures on birthweight by gestational age. And some indicators of newborns (such as APGAR scores and NICU rate) were not included in our follow-up database, which needs to be improved in the future. Moreover, some meaningful variables (such as the duration of embryo cryopreservation and perinatal outcomes of fresh embryo transfer cycles) were not included in this study, which may also create a bias. Nevertheless, the present study still presented perinatal outcomes of singletons following double vitrification-warming procedures, which are occasional in the clinic, providing valuable information for clinical decision-making.

## Conclusions

In summary, our results presented the neonatal safety of human refrozen-thawed embryos. Double vitrification-warming procedures of human embryos at different developmental stages do not affect perinatal outcomes. The re-cryopreservation procedure would be a valuable option to increase the cumulative pregnancy rate while preventing embryo waste under full informed consent. However, the maternal and child safety of the re-cryopreservation procedure still needs to be confirmed by a long-term multicentre follow-up cohort study.

## Supplementary Information

**Additional file 1 Supplemental Table 1.** Maternal outcomes of once-vitrified group and re-vitrified group before and after PS matching.

## Data Availability

The datasets generated and/or analysed during the current study are not publicly available for legal or ethical reasons, but are available from the corresponding author on reasonable request.

## Abbreviations

FET: Frozen-thawed embryo transfer
PSM: Propensity score matching
PTB: Preterm birth
LBW: Low birthweight
LGA: Large for gestational age
SGA: Small for gestational age
IVF: In-vitro fertilization
ICSI: Intracytoplasmic sperm injection
BMI: Body mass index
FSH: Follicle-stimulating hormone
LH: Luteinizing hormone
E2: Estradiol
HRT: Hormone-replacement therapy
HCG: Human chorionic gonadotropin
IQR: Interquartile range
OR: Odds ratio
ART: Assisted reproductive technology
GDM: Gestational diabetes mellitus

## References

[1] Zhang W, Xiao X, Zhang J, Wang W, Wu J (2018). Clinical outcomes of frozen embryo versus fresh embryo transfer following in vitro fertilization: a meta-analysis of randomized controlled trials. Arch Gynecol Obstet.

[2] Zhang X, Ma C, Wu Z, Tao L, Li R (2018). Frozen-thawed embryo transfer cycles have a lower incidence of ectopic pregnancy compared with fresh embryo transfer cycles. Reprod Sci.

[3] Liu X, Bai H, Shi W, Shi J (2019). Frozen-thawed embryo transfer is better than fresh embryo transfer in GnRH antagonist cycle in women with 3-10 oocytes retrieved: a retrospective cohort study. Arch Gynecol Obstet.

[4] Wong KM, Mastenbroek S, Repping S (2014). Cryopreservation of human embryos and its contribution to in vitro fertilization success rates. Fertil Steril.

[5] Smith A, Tilling K, Lawlor DA, Nelson SM (2019). Live birth rates and perinatal outcomes when all embryos are frozen compared with conventional fresh and frozen embryo transfer: a cohort study of 337,148 in vitro fertilisation cycles. BMC Med.

[6] Maheshwari A, Pandey S, Amalraj Raja E, Shetty A, Hamilton M (2018). Is frozen embryo transfer better for mothers and babies? Can cumulative meta-analysis provide a definitive answer?. Hum Reprod Update.

[7] Litzky JF, Boulet SL, Esfandiari N, Zhang Y, Kissin DM (2018). Effect of frozen/thawed embryo transfer on birthweight, macrosomia, and low birthweight rates in US singleton infants. Am J Obstet Gynecol.

[8] Cutting R (2018). Single embryo transfer for all. Best Pract Res Clin Obstet Gynaecol.

[9] Zheng X, Chen Y, Yan J, Wu Y, Zhuang X (2017). Effect of repeated cryopreservation on human embryo developmental potential. Reprod BioMed Online.

[10] Farhi J, Elizur S, Yonish M, Seidman DS, Shulman A (2019). Assessment of a double freezing approach in the management of surplus embryos in IVF. Reprod BioMed Online.

[11] Jing M, Lin C, Zhu W, Tu X, Chen Q (2020). Cost-effectiveness analysis of GnRH-agonist long-protocol and GnRH-antagonist protocol for in vitro fertilization. Sci Rep.

[12] Gardner DK, Schoolcraft WB (1999). Culture and transfer of human blastocysts. Curr Opin Obstet Gynecol.

[13] Zhu L, Wang J, Chen L, Jiang W, Fang J (2022). Blastocyst development rate influences singleton gestational age of similarly graded blastocysts after vitrified-warmed single embryo transfer cycles. Reprod BioMed Online.

[14] Zhu L, Zhang R, Zhang S, Shi W, Yan W (2015). Chinese neonatal birth weight curve for different gestational age. Zhonghua Er Ke Za Zhi.

[15] Sha T, Yin X, Cheng W, Massey IY (2018). Pregnancy-related complications and perinatal outcomes resulting from transfer of cryopreserved versus fresh embryos in vitro fertilization: a meta-analysis. Fertil Steril.

[16] Yokota Y, Yokota H, Yokota M, Sato S, Araki Y (2001). Birth of healthy twins from in vitro development of human refrozen embryos. Fertil Steril.

[17] Farhat M, Zentner B, Lossos F, Bdolah Y, Holtzer H (2001). Successful pregnancy following replacement of embryos previously refrozen at blastocyst stage: case report. Hum Reprod.

[18] Smith LK, Roots EH, Dorsett MJ (2005). Live birth of a normal healthy baby after a frozen embryo transfer with blastocysts that were frozen and thawed twice. Fertil Steril.

[19] Koch J, Costello MF, Chapman MG, Kilani S (2011). Twice-frozen embryos are no detriment to pregnancy success: a retrospective comparative study. Fertil Steril.

[20] Sills ES, Murray GU, Genton MG, Walsh DJ, Coull GD (2009). Clinical features and reproductive outcomes for embryos undergoing dual freeze-thaw sequences followed by blastocyst transfer: critique of 14 consecutive cases in IVF. Fertil Steril.

[21] Wang M, Jiang J, Xi Q, Li D, Ren X (2021). Repeated cryopreservation process impairs embryo implantation potential but does not affect neonatal outcomes. Reprod BioMed Online.

[22] Murakami M, Egashira A, Murakami K, Araki Y, Kuramoto T (2011). Perinatal outcome of twice-frozen-thawed embryo transfers: a clinical follow-up study. Fertil Steril.

[23] Keane KN, Mustafa KB, Hinchliffe P, Conceicao J, Yovich JL (2016). Higher beta-HCG concentrations and higher birthweights ensue from single vitrified embryo transfers. Reprod BioMed Online.

[24] Opdahl S, Henningsen AA, Tiitinen A, Bergh C, Pinborg A (2015). Risk of hypertensive disorders in pregnancies following assisted reproductive technology: a cohort study from the CoNARTaS group. Hum Reprod.

[25] H. Petersen S, Westvik-Johari K, Spangmose AL, Pinborg A, Romundstad LB, et al. Risk of hypertensive disorders in pregnancy after fresh and frozen embryo transfer in assisted reproduction: a population-based cohort study with within-Sibship analysis. Hypertension. 2022. 10.1161/HYPERTENSIONAHA.122.19689 36154568

[26] Shaw L, Sneddon SF, Brison DR, Kimber SJ (2012). Comparison of gene expression in fresh and frozen-thawed human preimplantation embryos. Reproduction.

[27] Bortoletto P, Prabhu M, Baker VL (2022). Association between programmed frozen embryo transfer and hypertensive disorders of pregnancy. Fertil Steril.

[28] Shavit T, Oron G, Weon-Young S, Holzer H, Tulandi T (2017). Vitrified-warmed single-embryo transfers may be associated with increased maternal complications compared with fresh single-embryo transfers. Reprod BioMed Online.

[29] Ashrafi M, Gosili R, Hosseini R, Arabipoor A, Ahmadi J (2014). Risk of gestational diabetes mellitus in patients undergoing assisted reproductive techniques. Eur J Obstet Gynecol Reprod Biol.

[30] Wang J, Liu Q, Deng B, Chen F, Liu X (2021). Pregnancy outcomes of Chinese women undergoing IVF with embryonic cryopreservation as compared to natural conception. BMC Pregnancy Childbirth.

[31] Liu Y, Li N, An H, Li Z, Zhang L (2021). Impact of gestational hypertension and preeclampsia on low birthweight and small-for-gestational-age infants in China: a large prospective cohort study. J Clin Hypertens (Greenwich).

[32] Ye W, Luo C, Huang J, Li C, Liu Z (2022). Gestational diabetes mellitus and adverse pregnancy outcomes: systematic review and meta-analysis. BMJ.

[33] Kc K, Shakya S, Zhang H (2015). Gestational diabetes mellitus and macrosomia: a literature review. Ann Nutr Metab.

