# Correction: Perinatal outcomes of singletons following double vitrification-warming procedures: a retrospective study using propensity score analysis

**DOI:** 10.1186/s12884-023-05413-y

**Published:** 2023-02-11

**Authors:** Xiaoyue Shen, Min Ding, Yuan Yan, Chenyang Huang, Shanshan Wang, Jianjun Zhou, Jun Xing

**Affiliations:** Reproductive Medicine Center, Drum Tower Hospital Affiliated to Nanjing University Medical School, Zhongshan Road 321, Nanjing, 210008 China

**Correction: BMC Pregnancy Childbirth 23, 30 (2023)**

**https://doi.org/10.1186/s12884-023-05369-z**

Following publication of the original article [1], the authors identified an error in Fig. 1. The correct figure is given below.

**Fig. 1:**
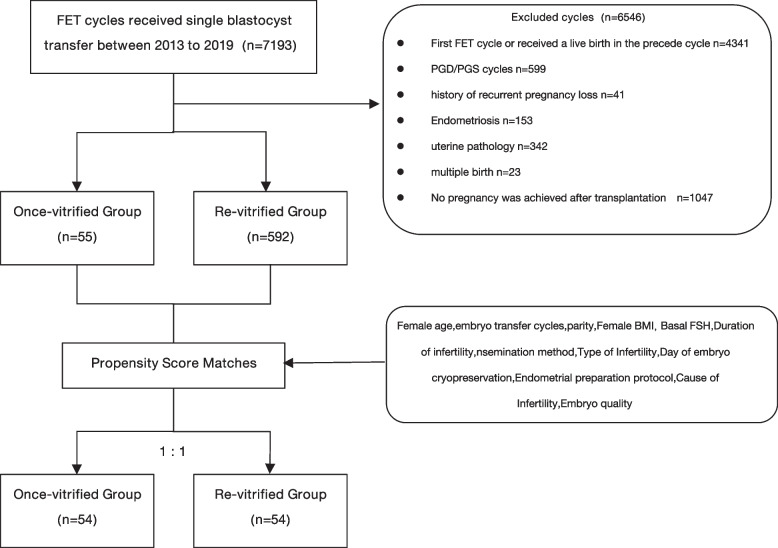
Flowchart of the inclusion and exclusion of participants in this study
