# Supplementary material for: Perinatal outcomes of singletons following double vitrification-warming procedures: a retrospective study using propensity score analysis
Source: BMC Pregnancy Childbirth. 2023 Jan 14;23:30. doi: 10.1186/s12884-023-05369-z (PMC9840308; doi:10.1186/s12884-023-05369-z)
Supplement: Supplementary file 1 — Additional file 1 Supplemental Table 1. Maternal outcomes of once-vitrified group and re-vitrified group before and after PS matching. [file 12884_2023_5369_MOESM1_ESM.docx]

**Supplemental Table 1 Maternal outcomes of once-vitrified group and re-vitrified group before and after PS matching.**

|  | Before matching | | | After matching | | |
| --- | --- | --- | --- | --- | --- | --- |
| Maternal complications | Once-vitrified group  （n = 592） | Re-vitrified group  （n = 55） | P | Once-vitrified group（n = 54） | Re-vitrified group  （n = 54） | P |
| Overall, n (%) | 98 (16.6) | 11 (20) | 0.51 | 7 (12.9) | 11 (20.4) | 0.46 |
| Gestational diabetes mellitus, n (%) | 52 (8.8) | 7 (12.7) | 0.33 | 4 (7.4) | 7 (12.9) | 0.53 |
| hypertensive disorders of pregnancy, n (%) | 32 (5.4) | 4 (7.3) | 0.53 | 4 (7.4) | 4 (7.4) | 1.00 |
| Placenta previa, n (%) | 13 (2.2) | 1 (1.82) | 1.00 | 1 (1.85) | 1 (1.85) | 1.00 |
| Placental abruption, n (%) | 1 (0.17) | 1 (1.82) | 0.16 | 0 (0) | 1 (1.85) | 1.00 |
